# Should psychomotor disturbance be an essential criterion for a DSM-5 diagnosis of melancholia?

**DOI:** 10.1186/1471-244X-13-160

**Published:** 2013-05-31

**Authors:** John Snowdon

**Affiliations:** 1 Discipline of Psychological Medicine, University of Sydney, Concord Hospital, Sydeny, NSW 2139, Australia

**Keywords:** Depression, Melancholia, Psychotic depression, Rating scales, Psychomotor retardation, Diagnostic classification systems, Aged

## Abstract

**Background:**

The CORE measure has proved useful in rating observed psychomotor disturbance (PMD), which has been held to be a key feature of melancholic depression. However, studies have shown a substantial percentage of subjects fulfilling DSM criteria for melancholia do not have observable PMD.

**Methods:**

A semi-structured interview schedule was used in assessing and diagnosing depressed older patients. DSM-IV diagnoses were made, and the CORE measure was used to rate PMD. Comparisons were made between melancholia inpatients who scored low and those scoring high on the CORE in relation to presentation and pattern of symptoms.

**Results:**

Of 32 inpatients with melancholia, 10 scored 0–7, 8 scored 8–10, and 14 scored 15 or more on the CORE. Thirty-two inpatients with psychotic depression scored 13 or more. High-CORE participants manifested unvarying depression more often than did low-CORE participants, and were less likely to state that stress precipitated their depressive episode.

**Conclusions:**

High-CORE melancholia cases appear to have more in common with psychotic depression than do low-CORE cases. Designation of observable PMD as an essential criterion in making a diagnosis of melancholia could increase the utility of the DSM classification in relation to treatment planning.

## Background

DSM-5 is to be published in mid-2013. Those responsible plan to keep the section relating to melancholia in DSM-5 unchanged from what was included in DSM-IV [1]. As in DSM-IV, if relevant criteria are fulfilled, the specifiers “with melancholic features” and “severe, with psychotic features” will be applicable in cases of major depression and bipolar disorder.

Various experts have argued for “reinstatement” of melancholia as a defined mood disorder in psychiatric classification [2]. They have declared that “melancholia should be acknowledged as an entity, not marginalized as a secondary specifier” [3]. In their view, “melancholia is a distinctive syndrome clinically defined by specific and pervasive disturbances in affect, psychomotor activity, vegetative functions, and cognition – and, in a subset of patients, by psychosis”. Note that their concept of melancholia subsumes cases of psychotic depression.

DSM-5 will allow for melancholia being recognised as an entity, but this will require clinicians and researchers to document, in each case where DSM-5 criteria for major depression with melancholic features are fulfilled, that this is the diagnosis. People with psychotic depression will usually fulfil criteria for melancholia as well as manifesting psychotic features, and for them the preferred label might be “melancholia with psychotic features”. Those diagnosed as having major depression (without using a specifier) would be presumed not to have melancholia or psychosis. Clinicians who diagnose melancholia can use guidelines and research evidence relating to melancholia when planning treatment and predicting prognosis.

The other major concern, highlighted by experts such as Fink and Taylor [4], Parker [5,6], and others, is that current DSM criteria for melancholia [1] do not provide adequate delineation between major depression with and without melancholic features. Parker [7] noted that endogeneity symptom scores of people with major depression showed a unimodal distribution and failed to distinguish melancholic and nonmelancholic patterns.

The feature found most consistently to differentiate melancholia from other types of depressive disorder is psychomotor change [8-10]. Sobin and Sackheim [11] declared that psychomotor symptoms “may be the only symptoms of depression that distinguish depression subtypes, and are predictive of good response to tricyclics antidepressants”. Parker [5] contrasted the marked specificity of observable psychomotor disturbance (PMD) in melancholic depression against the more ubiquitous distribution of endogeneity symptoms in cases of major depression. His group has developed the CORE measure [12] to rate degrees of PMD. Their findings led them to suggest that PMD is both necessary and sufficient to the definition of melancholia [7]. Schrijvers et al. [13] commented that “more and more evidence links psychomotor disturbances to the (psychotic and non psychotic) melancholic subtype”. They suggested that recent findings “might justify an elimination of the psychomotor item from the MDE core symptom list, leaving it exclusively as a key symptom of the melancholic subtype”.

The present author (JS) works in old age psychiatry, using the CORE measure and depression rating scales when assessing depressed older patients and their response to interventions, and referring to DSM criteria when making diagnostic decisions. His doctoral thesis provided some of the data set out below. Over the years he has seen a number of patients who undoubtedly fulfilled DSM criteria for major depression with melancholic features, but who manifested little or no PMD, even though, in many cases, the severity of depression as rated on the Brief Assessment Scale [14] and Hamilton Depression Rating Scale [15] was as high as in cases with marked psychomotor changes. Because of a paucity of comparisons in psychiatric journals (and partly to provoke consideration of revising criteria for “with melancholic features” in DSM-5), a pilot examination of melancholia cases (a majority consecutive) in the author’s clinical files was conducted, to look for differences associated with presence or absence of PMD. It was recognised that the small size of the retrospective study would not allow definitive answers. Nevertheless, in the absence of larger studies, the findings could prompt others to conduct adequately powered investigations.

A particular aim of the study was to assess whether cases with PMD were more likely than those without PMD to have had clinical manifestations and trajectories that were similar to cases of psychotic depression. Correspondingly, do the comparisons show evidence that cases of DSM-IV melancholia without PMD should be grouped with the non-melancholic depressions in spite of fulfilling 5 or more DSM criteria for melancholia?

## Method

Data sheets from subjects examined by JS in studies of depressed patients aged 65 years or older, admitted to psychiatric units, were re-examined. The methodology of his doctoral study has been presented previously [16]. In brief, consecutively referred patients were seen by JS, who used a semi-structured interview schedule devised for the study [17] to enquire about personal, psychiatric and medical history, plus recent psychiatric and physical symptoms. Three quarters of the assessed patients were under the clinical care of specialists other than JS. Stress was recorded as likely to have been a precipitant factor if participants or informants provided details concerning markedly anxiety-provoking pre-onset circumstances. JS recorded ratings on the 17-item Hamilton Depression Rating Scale (HDRS-17) [15], Brief Assessment Schedule (BAS) [14], and Mini-Mental State Examination (MMSE) [18], and on the CORE measure [[12]; see Appendix], an 18-item observer-rated scale used to rate retardation, agitation and cognitive processing problems. The CORE has proved superior to comparable tools in its potential to differentiate melancholia from other types of depression, in regards to illness variables, psychosocial risk factors, neuropsychological testing and treatment response [5].

Finally, JS made diagnostic assessments. Comparison with ratings and diagnoses made independently by other psychiatrists at Mood Disorder Unit meetings to co-rate patients showed satisfactory reliability [16,17]. Further details were obtained from hospital files & observations. Discharge summaries provided the final diagnoses made by the clinically responsible specialists. The analysis was extended to include other cases where the same protocol was used.

For the current analysis, findings from those diagnosed as fulfilling criteria for DSM-IV Major Depressive Episode with Psychotic Features or with Melancholic Features (i.e. melancholia) were examined. Co-morbid physical illnesses did not lead to exclusion providing the referred patients fulfilled the above criteria. In-patients with MMSE scores less than 24 were excluded, and four with bipolar disorder were considered separately. Of the remaining 64 subjects, 32 were diagnosed psychotic, 32 melancholic.

**Figure 1:**
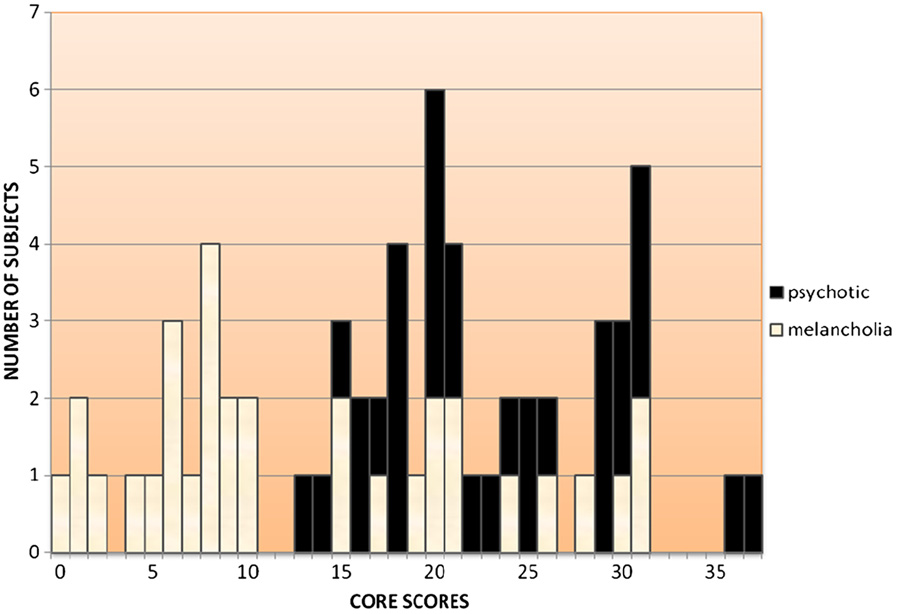
CORE scores of non-bipolar inpatients diagnosed as having DSM-IV melancholia or psychotic depression.

There appeared to be a biphasic pattern to the CORE scores of melancholia subjects, one group scoring 0 to 10, the other 15 to 31 (Figure 1), and therefore it was decided to compare these two groups rather than impose the 7/8 cut-point derived by Parker and colleagues [9] in their studies across the age-range. However, results were examined to see if using 7/8 as a cut-point rather than 10/11 made any difference to derived conclusions. Comparisons were made between the two melancholia groups, and between melancholia and psychotic depression groups, in relation to psychiatric symptoms, and history of depressive episodes and treatment. A comparison between the total groups of participants with psychotic depression, melancholia (with and without PMD) and those with nonmelancholic depression has been provided elsewhere [16].

Finally, for each participant who scored 10 or less on the CORE, note was made concerning ratings of the presence of symptoms that are included as criteria for DSM-IV melancholia [1]: (1) lost pleasure in activities, (2) lost reactivity to pleasurable stimuli, (3) distinct quality of depressed mood, (4) depression worse in the morning, (5) early morning waking, (6) marked psychomotor retardation or agitation, (7) significant anorexia or weight loss, and (8) excessive or inappropriate guilt. Note was made of whether participants expressed feelings of being slowed up, and of the HDRS-17 ratings of retardation and agitation made at the time.

Approval for this study was provided by the ethics committee of the Eastern Sydney Area Health Service.

## Results

The CORE scores of the 32 identified unipolar melancholia subjects are represented in Figure 1, ten scoring 0 to 7, eight scoring 8 to 10, and fourteen scoring 15 to 31. Scores of 32 inpatients with psychotic depression are also shown in Figure 1. These can be compared with mean CORE scores of participants in JS’s doctoral study of late-life depression [16]: psychotic depression 22.0 (s.d. 8.3), melancholia 12.9 (s.d. 9.3), and non-melancholic depression 6.4 (s.d. 5.1).

A comparison of demographic factors, history of their depression, and scores on rating scales, between low-CORE (score 0 to 10) and high-CORE (15 to 31) melancholia subjects is shown in Table 1, together with percentages of those rated as having certain symptoms (where there appeared to be some difference between groups). The Chi square test was used to examine the significance of differences in the frequency of depressive features in the three groups. The low-CORE and high-CORE groups were too small to expect to find markedly significant differences. However, inclusion of inpatients with psychotic depression (all high-CORE) demonstrates patterns of difference across Table 1, from low-CORE through high-CORE melancholia to psychotic depression, in ratings of diurnal variation, unvarying depression and feeling that punishment is deserved.

**Table 1:** DSM-IV melancholia (low-CORE, high-CORE) inpatients compared to inpatients with psychotic depression (CORE scores >12)

	**Melancholia CORE 0 to 10 (N = 18)**	**Melancholia CORE 15 to 31 (N = 14)**	**Psychotic CORE 13 to 37 (n = 32)**
**Sex ratio**	**14 F : 4 M**	**9 F : 5 M**	**18 F : 14 M**
**Age (mean)**	**74.7 years**	**77.4 years**	**75.0 years**
**Age range**	**69**–**85 years**	**67**–**91 years**	**65**–**87 years**
**Age of onset (mean)**	**49.8 years**	**65.4 years**	**56.2 years**
**Onset age 65+**	**41%**	**79%**	**53%**
**Duration (mean)**	**35 weeks**	**49 weeks**	**>50 weeks**
**Hamilton-17 (mean)**	**25.9**	**29.1**	**27.8**
**BAS (mean)**	**17.2**	**18.6**	17.4*
**MMSE**	**26.6**	**26.9**	26.7*
**Had depression previously**	**89%**	**64%**	**77%**
**Had antidepressants before**	**78%**	**64%**	**77%**
**Had E.C.T. previously**	**67%**	**50%**	**68%**
**Stress as precipitant**	**89% +**	**57%**	**52%**
**Diurnal variation (worse in a.m.: moderate/severe)**	**61% +**	**36%**	**10%**
**Unvarying depression**	**22% +**	**79%**	**100%**
**Worthlessness**	**61%** **+ (4/10 with score <8; 7/8 with score 8–10)**	**86%**	**74%**
**Punishment felt to be deserved**	**0%**	**29% (4/14)**	**52%**

* Data incomplete re BAS and MMSE for 4 inpatients with psychotic depression, but their MMSE scores were measured as >24 on discharge from hospital.

**+ **χ^2 ^tests comparing low-CORE and high-CORE groups showed significant differences: **Stress as precipitant **(χ^2^ = 6.70, p < .01), and **Unvarying depression **(χ^2^ = 7.91, p < .01), but not for **Diurnal variation **(χ^2^ = 1.14) or **Worthlessness **(χ^2^ = 1.30).

Table 2 shows that all low-CORE subjects would have fulfilled criteria for DSM-IV melancholia, though three (having CORE scores of 1, 6 and 7) would not have done so if there had been a requirement (as in DSM-III-R) that the marked psychomotor retardation or agitation be observed as well as experienced. The mean combined score on the HDRS-17 agitation and retardation items in the DSM-IV melancholia group scoring 0 to 10 on the CORE was 1.0, whereas for the group scoring 15 to 31 it was 3.0, the HDRS-17 total scores for these two groups being, respectively, 25.9 (range 16–29) and 29.1 (range 22–32). The correlation between CORE and HDRS-17 scores in the late-life depression study [16] was quite high (r = .63), unlike that between CORE and BAS ratings (r = .065); PMD ratings do not count towards BAS scores.

**Table 2:** Number of DSM-IV melancholia criteria fulfilled and HDRS-17 ratings in low-CORE inpatient cases

	**CORE score**	**Number of DSM-IV melancholia criteria fulfilled**	**Feelings of being slowed**	**Hamilton rating of retardation**	**Hamilton rating of agitation**
**F 80**	**0**	**8**	**Yes**	**0**	**0**
**F 71**	**1**	**5**	**Yes**	**0**	**0**
**F 83**	**1**	**5**	**Yes**	**0**	**1**
**F 74**	**2**	**7**	**Yes**	**0**	**0**
**F 68**	**4**	**6**	**Unsure**	**1**	**0**
**M 75**	**5**	**6**	**Unrecorded**	**1**	**0**
**F 82**	**6**	**7**	**Yes**	**1**	**1**
**M 77**	**6**	**5**	**Yes**	**0**	**0**
**F 79**	**6**	**6**	**Unsure**	**1**	**1**
**F 69**	**7**	**5**	**Yes**	**0**	**0**
**M 67**	**8**	**8**	**Yes**	**0**	**0**
**F 69**	**8**	**6**	**Yes**	**1**	**0**
**F 76**	**8**	**8**	**Yes**	**1**	**1**
**F 70**	**8**	**7**	**Yes**	**2**	**0**
**F 77**	**9**	**6**	**Yes**	**2**	**1**
**F 78**	**9**	**6**	**Yes**	**1**	**0**
**F 73**	**10**	**7**	**Yes**	**1**	**3**
**M 84**	**10**	**6**	**Yes**	**1**	**0**

Of the 24 in-patients who were under the care of other specialists but diagnosed by the author as fulfilling DSM-IV criteria for Major Depression with Melancholic Features, 15 were recorded on discharge summaries as having Major Depression with melancholia, or agitated depression. The other 9 were discharged with labels of Major Depression (6), Recurrent Depression (1), Severe Depression (1), or Depression not otherwise specified (1). The time elapsing between assessment by JS and hospital discharge (with documentation of a final diagnosis) varied between 1 and 140 days. Note that none was diagnosed as showing psychotic features.

Four other participants with melancholic and/or psychotic features and no evidence of organic brain disease were not included in the above analysis. They had histories diagnostic of bipolar disorder. All had their first episode of mood disorder in their forties, and all had been given ECT at some time. One (non-psychotic melancholic) had a CORE score of 0, but a BAS score of 18 (markedly depressed). The other participant with melancholia but without psychotic features had a CORE score of 14 and BAS score of 15. Both bipolar patients with psychotic features had CORE scores of 25 and high depression scores (mean HDRS score = 23). Adding their scores to those included in Table 1 did not change the significance of any of the differences displayed.

## Discussion

There is a need for further examination of the criteria used for defining melancholia. Parker [5] and colleagues recommended that PMD be designated a required feature.

This study has shown that most of a high-CORE melancholia group and all those with psychotic depression described depression that did not vary in the day or between days, whereas there was a trend for low-CORE subjects to experience moderate or marked diurnal variation more often. The high-CORE group had higher mean HDRS scores, but this is partly because (as shown in Table 2) retardation and agitation ratings contribute to HDRS totals. Stress was significantly more often a precipitant of low-CORE melancholia, while there was a trend for worthlessness to be more often expressed by high-CORE subjects, and for onset to have been later in life in high-CORE melancholia cases. Half the depressive psychosis inpatients, over one quarter of the high-CORE melancholia subjects and none of the low-CORE subjects believed they deserved punishment. As previously reported [16], this belief, together with PMD, hallucinations, delusions and the absence of diurnal mood variation, are features on which cases of psychotic depression differ most distinctly from those of melancholia.

Snowdon [17] used factor analysis to develop a new scale, with items that included decision difficulty, poor concentration, irritability and brittle temper, and found that low-CORE non-psychotic melancholic patients scored as “more neurotic” than high-CORE participants. They also showed more “negative cognition” – helplessness, hopelessness, pessimism and poor life-satisfaction.

From these differences, it could be hypothesised that low-CORE melancholia is more likely to be related to stress and personality characteristics, whereas high-CORE melancholia may be related to biological factors.

Although Parker and colleagues [12] have argued for the differentiation of melancholia from psychotic depression, the finding that psychosis is often present in cases of melancholia [12] could facilitate a compromise approach. DSM-5 could specify that persons with Major Depression plus PMD and/or psychotic symptoms not attributable to an organic disease should be specified as having melancholia with or without psychotic features. Those without either PMD or psychotic symptoms would be considered to have Major Depression (and melancholia would not be specified).

The DSM-IV specifier for melancholic features requires that five out of the eight criteria must be fulfilled, but not necessarily the PMD criterion. As shown in Figure 1, there appeared in this study to be two distinct groups of melancholia cases, one with low (0–10) and one with high (15–31) CORE scores. All participants were elderly, in contrast to most of those included in early studies using the CORE, in which 7/8 was recommended as the cut-off. A cut-off of 10/11 was used in the present study when analysing data. Age-related adjustments regarding normative data may be as appropriate for the CORE as they are for the MMSE [18]. Researchers assessing French inpatients with depression (mean age 54.7 years) found a cut-off score of 5/6 most appropriate, but noted that none had psychotic depression [19].

Some studies of outpatients with melancholia have shown absence of PMD to be comparatively common. Benazzi [20] found PMD in only 35.4% of 48 outpatient cases with melancholia (mean age 47 years, 56% having Bipolar-II Disorder). New Zealand researchers [21] reported that 66% of outpatients fulfilling DSM-IV melancholia criteria (mean age 32.5 years) scored 8 or more on the CORE. An earlier cross-age Australian study, including bipolar cases, showed 54% of patients with DSM-III-R melancholia scored above the same cut-off [9].

A limitation of this study is that findings from an old age psychiatry service cannot be generalised to younger adult populations. None of the participants had co-morbid dementia, but a number (particularly of those with psychotic depression, as shown in Table 1) had late-onset depression, raising the possibility that they had vascular depression [22]. PMD is commonly a feature of such cases, in contrast to early-onset cases. Total CORE scores have been shown to increase with age, particularly among depressed people with melancholia [23,24]. A second limitation of the study is that neuroimaging results were unavailable in many of the earlier cases.

A further limitation is that the DSM-IV diagnoses of depressive disorder were made by one clinician (JS), though it was reassuring to note that discharge diagnoses made by their own psychiatrists were in a majority of cases identical to those of JS. In some cases (see Results), these psychiatrists had diagnosed depression but not used the DSM classification, though the files highlighted melancholic manifestations.

Fourthly, the number of participants with melancholia but without psychotic features was too small for detailed analysis concerning sub-groups.

## Conclusion

The pilot study described in this paper was small. It points to differences between DSM-IV melancholia subjects with and without observed PMD. A larger study of variables and outcome in relation to cases of depression that fulfil current criteria for melancholia, with and without observable PMD, is needed. It would help determine whether PMD should be an essential criterion for specifying the presence of psychotic and/or melancholic features, and (in due course) whether diagnostic categories of Melancholic and/or Psychotic Depression should be established and coded. Taylor and Fink [10] declared that giving melancholia a separate category (psychomotor disturbance being an essential criterion) “will increase its recognition and encourage the use of the most appropriate treatments”.

## Appendix

The CORE measure [Parker and colleagues; [12]]

Each item is rated on a 0–3 scale

Non-interactiveness items: 1, 4, 7, 8, 12, 16

Retardation items: 2, 3, 6, 10, 13, 15, 17

Agitation items: 5, 9, 11, 14, 18

1. Non-interactiveness (no response to social cues; fails to interact with interviewer)

2. Facial immobility (lack of fluctuation of facial expression)

3. Postural slumping (head bowed, shoulders rolled forwards)

4. Non-reactivity (in response to attempts to cheer them up)

5. Facial apprehension (perplexity, tortured concern, etc.)

6. Delay in responding verbally

7. Length of verbal responses

8. Inattentiveness (impaired concentration)

9. Facial agitation (facial movements indicate fear, anguish, torment)

10. Body immobility (amount, not speed)

11. Motor agitation (cannot stay still: rubbing, pacing, writhing)

12. Poverty of associations (vagueness; themes lack explication)

13. Slowed movement (speed, not amount)

14. Verbal stereotypy (speech repetitive, on limited themes)

15. Delay in motor activity

16. Impaired spontaneity of talk

17. Slowing of speech rate

18. Stereotyped movements (repetitive or purposeless)

## Competing interests

The author has no competing interests to declare, in relation to this article.

## Pre-publication history

The pre-publication history for this paper can be accessed here:

http://www.biomedcentral.com/1471-244X/13/160/prepub
